# CD16 (FcRγIII) as a potential marker of osteoclast precursors in psoriatic arthritis

**DOI:** 10.1186/ar2915

**Published:** 2010-01-26

**Authors:** Yahui Grace Chiu, Tianmeng Shao, Changyong Feng, Kofi A Mensah, Michael Thullen, Edward M Schwarz, Christopher T Ritchlin

**Affiliations:** 1 Allergy/Immunology & Rheumatology Unit, University of Rochester Medical School, 601 Elmwood Avenue, Rochester, NY 14642, USA; 2 The Center for Musculoskeletal Research, University of Rochester Medical School, 601 Elmwood Avenue, Rochester, NY 14642, USA; 3 Analytical Biochemistry, MedImmune, LLC., One MedImmune Way, Gaithersburg, MD 20878, USA; 4 Department of Biostatistics, University of Rochester Medical School, 601 Elmwood Ave., Rochester, NY 14642, USA; 5 Department of Microbiology and Immunology, University of Rochester Medical School, 601 Elmwood Avenue, Rochester, NY 14642, USA

## Abstract

**Introduction:**

Psoriatic arthritis (PsA) is a chronic inflammatory arthritis characterized by bone erosion mediated by osteoclasts (OC). Our previous studies showed an elevated frequency of OC precursors (OCP) in PsA patients. Here, we examined if OC arise from CD16-positive monocytes in PsA.

**Methods:**

Peripheral blood mononuclear cells (PBMC) or monocytes were isolated from human peripheral blood and sorted based on CD16 expression. Sorted cells were cultured alone or with bone wafers in the presence of receptor activator of nuclear factor kappa-B ligand (RANKL) and macrophage colony-stimulating factor (M-CSF). Enumeration and bone erosion activity of OC were examined after culture. The effects of tumor necrosis factor-alpha (TNFα), OC-promoting (M-CSF plus RANKL), and dendritic cell (DC)-promoting (GM-CSF plus interleukin (IL)-4) cytokines on CD16 surface expression were examined by flow cytometry.

**Results:**

PsA and psoriasis (Ps) subjects had a higher percentage of circulating inflammatory CD14+CD16+ cells than healthy controls (HC). Exposure of cells to OC-promoting, but not DC-promoting media, was associated with CD16 up-regulation. PBMC of Ps and PsA had a higher frequency of cells expressing intermediate levels of CD16. OC were mainly derived from CD16+ cells in PsA. Increased CD16 expression was associated with a higher bone erosion activity in PsA.

**Conclusions:**

An increased frequency of circulating CD14+CD16+ cells was noted in PsA compared to controls, and intermediate levels of CD16 may suggest a transitional state of OCP during osteoclastogenesis. Intriguingly, TNFα blocked CD16 expression on a subset of CD14+ monocytes. Collectively, our data suggest that CD16 has the potential to serve as an OCP marker in inflammatory arthritis.

## Introduction

Psoriatic arthritis (PsA) is an inflammatory joint disease characterized by joint destruction in the majority of patients within two years of disease onset [1]. Joint damage is carried out by synovial fibroblastoid cells that degrade cartilage through the release of metalloproteinases and osteoclasts (OC), which directly resorb bone. OC are multinucleated cells that arise from osteoclast precursors (OCP) or circulating CD14+ monocytes through a differentiation process referred to as osteoclastogenesis [2]. Of particular interest in regards to PsA was the finding of an increased frequency of OCP in one-third of patients with psoriasis (Ps) without arthritis and in the majority of PsA patients [3]. Intriguingly, monocytes circulating in the peripheral blood of PsA patients were able to generate OC *in vitro *in the absence of exogenous stimulation [3], a property distinct from OCP in healthy controls (HC). Importantly, the frequency of OCP correlated with the extent of radiographic damage in a cohort of patients with established PsA [3]. Thus, identification of specific surface markers of OCP is of great interest, given that the current assessment of OCP requires laborious, expensive, and time-consuming cell culture.

For the current study, we chose CD16, the low-affinity immunoglobulin (Ig) G Fcγ receptor (FcγRIIIa), as a candidate cell surface marker of OCP for several reasons. First, the CD16+ human monocyte subset is considered 'pro-inflammatory' [4-6]. These cells exhibit several unique properties with characteristics of an OCP population. The CD16+ monocyte subset is rare in healthy controls [5], but is preferentially expanded two- to four-fold during infection or inflammation [5-10]. Moreover, the percentage of CD16+ cells (5 to 10%) in human peripheral blood monocytes falls into a reasonable range for the OCP population. Second, CD16+ cells are expanded in the circulation of patients with rheumatoid arthritis (RA) and they are present in rheumatoid synovial tissue [11]. Importantly, this population is also expanded in the circulation of patients with aseptic joint loosening and osteolysis [12]. Third, CD14+CD16+ cells release TNFα and IL-6, cytokines that can greatly potentiate osteoclastogenesis and activate OCs, respectively [13]. CD16 is an oligomeric complex composed of one Fc-binding α chain associated with homodimers or heterdimers of the T-cell receptor ζ (TCR-ζ) and the γ subunit of FcεRI (FcRγ) [14], and thus belongs to the family of the multichain immunorecognition receptors [15]. The presence of the immunoreceptor tyrosine-based activation motif (ITAM) in the FcRγ subunit of CD16 complex notably accentuates the role of CD16 in signaling [16,17].

Previously, we showed that PsA patients have an elevated frequency of circulating OCP in their peripheral blood [3]. Based on the properties of the CD14+CD16+ population outlined above, we hypothesized that OCP in PsA arise from the CD16+ monocyte subset and thus, the CD16 molecule might serve as an OCP marker in PsA. To this end, we examined the expression of the CD16 molecule in a cohort of HC, Ps, RA, and PsA patients. We also examined the relation between CD16 expression, osteoclastogenesis potential and bone erosion activity.

## Materials and methods

### Study population

All clinical studies were carried out with the approval of the University of Rochester Medical Center Research Subjects Review Board and with informed consent. PsA was diagnosed according to the Moll and Wright Criteria [18]. Subjects with inflammatory arthritis were recruited from the faculty clinics at the University of Rochester Medical Center and Ps subjects from our Psoriasis Center. HCs had no acute or chronic joint pain and were in good health. None of the patients or controls was taking medication.

### Cell isolation

Peripheral blood mononuclear cells (PBMC) were separated from the whole blood by Ficoll gradient. Briefly, the blood sample was first diluted twice in sterile PBS and overlaid onto Ficoll-Paque PLUS (GE Healthcare, Piscataway, NJ, USA), soft spun at 2250 rpm for 45 minutes at room temperature without brake. PBMC were collected at the plasma/ficoll interface and washed once with PBS. Erythrocytes were lysed with ACK lysing buffer (BioWhittaker, Walkersville, MD, USA) and the remaining cells were washed twice with PBS in preparation for culture.

### Monocyte enrichment

Human monocytes were enriched by either positive or negative selection. Positive selection was performed with the CD14 MicroBeads (Miltenyi Biotec, Auburn, CA, USA) that bind to CD14 on the surface of monocytes. Negative selection was performed with the Human Monocyte Enrichment Cocktail (StemCell, Vancouver, Canada) that depletes lymphocytes by rosetting but leaves monocytes untouched. Both enrichment protocols were performed following manufacturers' instructions.

### Reagents and antibodies

Antibodies were purchased from BD Bioscience (San Jose, CA, USA) except the FITC-conjugated anti-MHCII (DP/DQ/DR) antibody (Ancell, Bayport, MN, USA). Receptor activator of nuclear factor kappa-B ligand (RANKL), macrophage colony-stimulating factor (M-CSF), and TNFα were purchased from R&D systems (Minneapolis, MN, USA). Defined fetal bovine serum was obtained from Hyclone (Logan, UT, USA).

### OC culture and TRAP staining

Purified PBMC or monocytes were cultured in RPMI (Gibco/Invitrogen, Grand Island, NY, USA), supplemented with 8% heat-inactivated fetal bovine serum, 2 mM glutamine, 50 units/ml penicillin, and 50 ug/ml streptomycin. RANKL (100 ng/ml) and M-CSF (25 ng/ml) were added to cell culture to stimulate OC generation. 1 × 10^5 ^of PBMC or monocytes (1 × 10^6 ^cells/ml) were cultured in one well of 96-well plate with triplicates in 5% carbon dioxide at 37°C. Media were replenished every two days. On day eight, cells were fixed with 3% formaldehyde and stained for tartrate-resistant acid phosphatase (TRAP; Sigma, St. Louis, MO, USA), and were examined by light microscope. TRAP+ cells with three or more nuclei were counted as OCs. To examine the effect of TNFα on CD16 expression, human PBMC were cultured in either plain media or media supplemented with TNFα (25 ng/ml) for three days, followed by antibody staining and flow cytometry analysis.

### Cell sorting and flow cytometry analysis

For sterile cell sorting, PBMC prepared from Ficoll gradient were re-suspended in sterile PBS at 10 × 10^6 ^cells/ml and incubated with anti-human CD16-PE (Clone 3G8, BD Bioscience, San Diego, CA, USA) and anti-human MHCII-FITC (Clone TDR31.1, Ancell, Bayport, MN, USA) antibodies for 30 minutes at 4°C. Cells were washed twice with PBS, re-suspended in PBS (5 × 10^6 ^cells/ml) and subjected to sterile sorting by FACS Vantage (Becton Dickinson Immunocytometry Systems, Bedford, MA, USA). After sorting, 1 × 10^5 ^cells were cultured in one well of a flat 96-well plate in the presence of RANKL and M-CSF for eight days, and TRAP-stained for OC enumeration. For flow cytometry analysis, cells were harvested, washed once with PBS, and stained with antibody cocktails for 30 minutes at 4°C. Cells were then washed twice with PBS, fixed, and analyzed by FACSCalibur, FACScanto, or LSRII with appropriate compensations. Flow data were analyzed by CellQuest software version 3.5.1 (Becton Dickinson Immunocytometry Systems, Bedford, MA, USA) or Flowjo software (Treestar, Ashland, OR, USA).

### Assessment of bone erosion via micro CT

Bone wafers were scanned via high-resolution *in vivo *micro-computed tomography (VivaCT40, Scanco, Southeastern, PA, USA) at an isotropic resolution of 17.5 m with 55 keV cone beam mode. The scanned data were reconstructed via Scanco software into Dicom files and further analyzed by the Amira 3.1 software. A density threshold of more than 11,000 AU was set in the SurfaceGen module to visualize the bone.

### Statistical analysis

**Figure 1:**
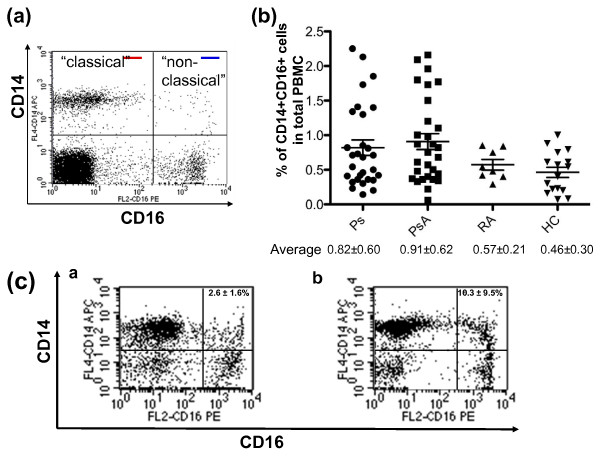
**Ps and PsA patients have a higher percentage of CD14+CD16+ cells**. Human peripheral blood mononuclear cells (PBMC) were isolated from peripheral blood, stained with an antibody cocktail composed of CD14-APC, CD16-PE, 7AAD, and analyzed by flow cytometry. Dead cells were excluded by 7AAD+. **(a) **Classical (CD14+CD16-, red line) and non-classical (CD14+CD16+, blue line) monocytes were labeled based on the classification by Strauss-Ayali and colleagues [5]. **(b) **The percentage of CD14+CD16+ cells in the PBMC of 16 healthy controls (HC), and 29 psoriasis (Ps), 28 psoriatic arthritis (PsA), and 8 rheumatoid arthritis (RA) patients. **(c) **The percentage of CD14+CD16+ cells in enriched human monocytes from HC and PsA. Monocytes were enriched by the Human Monocyte Enrichment Cocktail. The percentage of CD14+CD16+ cells in enriched monocytes from HC (2.6 ± 1.6%) and PsA patients (10.3 ± 9.5%) are shown in [a] and [b], respectively. The data are representative of 10 independent experiments.

One-way analysis of variance with Tukey's *post hoc *multiple comparison test was used to compare four different groups in Figure 1(b) and Table 1. The two-sample t-test was used for comparison of two groups with continuous data. The significance level was set at 0.05. The Satterthwaite two-sample test was used to analyze data presented in Figure 1(c). The Fisher's exact test was used to analyze the frequencies of CD16 expression presented in Table 1. All statistical analyses were performed on SAS 9.1 (SAS Institute Inc., Cary, NC, USA).

**Table 1:** Percentage of CD14+CD16+ cells in total PBMC and CD16^int ^cells in Ps, PsA, RA and HC populations

(a)	(b)		(c)
	**Number of subjects^b^**		**% of subject^c^**
		
**% of CD14+CD16+ in total PBMC^a^**	**with CD16^int^**	**without CD16^int^**	**with CD16^int^**

Ps (n = 29) 0.82 ± 0.60	25	4	86
PsA (n = 29) 0.91 ± 0.62	19	9	67
RA (n = 8) 0.57 ± 0.21	5	3	61
HC (n = 16) 0.46 ± 0.30	8	11	42

^a ^Total PBMC were purified by Ficoll gradient, stained with PE-CD16, allophycocyanin (APC)-CD14 antibodies and analyzed by flow cytometry. Dead cells were excluded by 7-amino-actinomycin D (AAD).

^b ^Number of subjects with or without CD16^int^. CD16^int ^cells.^c ^Percentage of subjects with CD16^int^.

HC = healthy control; PBMC = peripheral blood mononuclear cells; Ps = psoriasis; PsA = psoriatic arthritis; RA = rheumatoid arthritis.

## Results

### The CD14+CD16+ monocyte subset is increased in PsA patients

Based on CD16 expression and the monocyte classification criteria set by Strauss-Ayali and colleagues [5], human CD14+ monocytes can be divided into two subsets, classical and non-classical, as shown in Figure 1(a). Several studies have demonstrated that the pro-inflammatory, 'non-classical' CD14+CD16+ monocyte subset is expanded in many inflammatory diseases including RA [7,9,19]. Recently, the expansion of this subset was also found in patients with aseptic loosening [12]. However, the frequency of this unique monocyte subset in Ps and PsA subjects is unknown. To this purpose, we examined the percentage of CD14+CD16+ monocytes in a cohort of Ps (n = 29) and PsA (n = 29) subjects and compared the findings with those in HC (n = 16) and patients with RA (n = 8). It is important to note that none of these subjects were on disease modifying anti-rheumatic drugs (DMARDs) or anti-TNF medications.

As shown in Figure 1(b), the percentage of CD14+CD16+ cells in Ps, PsA, RA and HC were 0.82 ± 0.60%, 0.91 ± 0.62%, 0.57 ± 0.21%, and 0.46 ± 0.30%, respectively. The average percentage of CD14+CD16+ cells in Ps and PsA patients increased 1.8- to 2-fold over levels in HC. A significant difference was observed when PsA subjects were compared with controls (*P *= 0.04). However, it is noteworthy that the percentage of CD14+CD16+ cells varied considerably between individuals (shown by distributions of dots in Ps and PsA subjects, Figure 1(b)). These results are summarized in the (a) column of Table 1.

Based on the current concept that monocytes are the major reservoir of human OCP, we further analyzed the percentage of CD14+CD16+ cells on enriched monocytes (Figure 1(c)). The percentage of purified monocytes that expressed CD14+CD16+ was 2.6 ± 1.6% in HC (Figure 1(c)-a, n = 11), a number close to the result published by Ancuta and colleagues [20]. Notably, the percentage of CD14+CD16+ cells was significantly increased in PsA patients (Figure 1(c)-b, 10.3 ± 9.5%, n = 9; *P *= 0.04). Taken together, results from PBMC (Figure 1(b)) and enriched monocytes (Figure 1(c)) demonstrate a significant expansion of CD14+CD16+ cells in Ps and PsA patients.

### CD16 surface expression is up-regulated by osteoclastogenic cytokines

It is known that OC and myeloid dendritic cells (DC) are derived from the same hematopoietic monocytic progenitor cells [21]. Human monocytes mature into OC in the presence of RANKL and M-CSF, and differentiate into DC when exposed to IL-4 and granulocyte-macrophage colony-stimulating factor (GM-CSF) [22]. We postulated that the surface expression of CD16 on monocytes might be essential for OC development but not for DC differentiation. To test this hypothesis, we compared the effect of RANKL + M-CSF with GM-CSF + IL-4 on CD16 surface expression.

**Figure 2:**
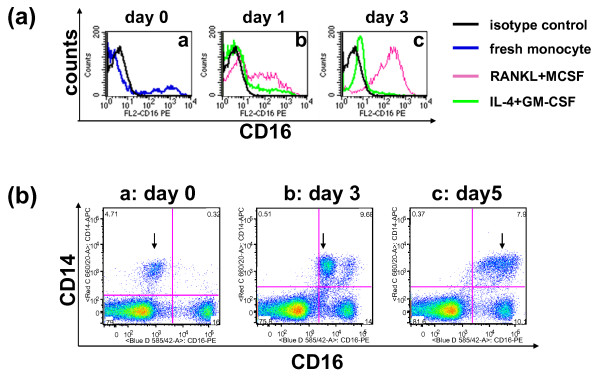
**Cytokines alter the cell surface expression of CD16 and human CD14+ cells undergo a transitional stage of CD16 up-regulation in OC-promoting culture conditions**. **(a) **Enriched human monocytes were cultured in osteoclast (OC)-promoting media (receptor activator of nuclear factor kappa-B ligand (RANKL) + macrophage colony-stimulating factor (M-CSF), pink line) or dendritic cell (DC)-promoting media (IL-4 + granulocyte-macrophage colony-stimulating factor (GM-CSF), green line). Freshly isolated monocytes (blue line) and the isotype control (black line) are also shown. Surface expression of CD16 was monitored by FACS analysis on [a] day 0, [b] day 1, and [c] day 3, respectively. **(b) **Human peripheral blood mononuclear cells (PBMC) were cultured in OC-promoting media (RANKL + M-CSF) and the cell surface expression of CD14 and CD16 was monitored on [a] day 0, [b] day 3, and [c] day 5. Data shown here are live cells after forward scatter/side scatter (FSC/SSC) gating followed by dead cell exclusion using 7-amino-actinomycin D (AAD). Numbers shown in each quadrant are the percentage of total gated cells.

The cell surface expression of CD16 was monitored at two different time points after monocytes were purified from PBMC and cultured in plain, OC-promoting (RANKL + M-CSF), or DC-promoting (GM-CSF + IL-4) media (Figure 2(a)). Anti-CD16-PE, anti-CD14-allophycocyanin (APC) antibodies and 7-amino-actinomycin D (AAD) were included in our antibody staining panel. PBMC was first gated using forward scatter/side scatter (FSC/SSC) followed by dead cell exclusion by 7-AAD. Freshly isolated human monocytes are heterogeneous in regard to CD16 surface expression, and the majority of cells are CD16- (blue line in Figure 2(a)-a). After 24 hours in culture, the expression of CD16 increased in all culture conditions (Figure 2(a)-b). A significant polarization of CD16 cell surface expression was observed when cells were cultured for a longer period of time. By day three (Figure 2(a)-c), CD16 surface expression decreased on cells cultured in IL4+GM-CSF (green line in Figure 2(a)-c), but increased on cells cultured in RANKL+M-CSF (pink line in Figure 2(a)-c). Our results showed that CD16 upregulation occurred when monocytes were driven into OC but not DC lineage.

Next, we examined the dynamic expression of CD16 in relation to CD14 during OC differentiation. The expression of CD16 and CD14 was monitored after PBMC were cultured in OC-promoting media (RANKL+M-CSF) on day 0, day 3, and day 5, respectively (Figures 2(b), a to 2c). Intriguingly, CD14+ monocytes showed a gradual increase of CD16 expression (shown by arrows in Figures 2(b), a-c). We identified a population that expressed an intermediate level of CD16 (arrow in Figure 2(b)-b) during the conversion of CD16- (arrow in Figure 2(b)-a) toward CD16+ (arrow in Figure 2(b)-c). These data indicated that OCPs may undergo a transitional differentiation stage (shown by arrow in Figure 2(b)-b) before they develop into mature OC and upregulate CD16 to a maximal level (Figure 2(b)-c).

### A subset of PBMC expresses intermediate levels of CD16

**Figure 3:**
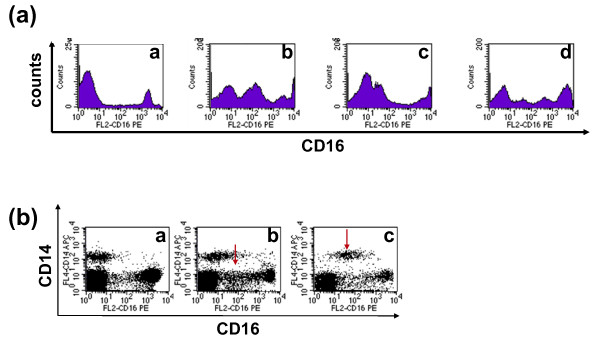
**Cells that express intermediate levels of CD16 (CD16^int^) are found *in vivo *in human PBMC**. **(a) **CD16 expression on human peripheral blood mononuclear cells (PBMC) is heterogeneous. The data shown here are the representatives of CD16 expression patterns on fresh human PBMC from 82 subjects. [a] PBMC that expressed high and low levels of CD16 without the CD16^int ^population. [b to d] PBMC that have CD16^int ^populations with different CD16 expression levels. **(b) ***Ex vivo *analysis of CD14 and CD16 expression. Representative examples of individuals [a] without a CD16^int ^population, [b] CD14- CD16^int ^(arrow) cells and [c] CD14+ CD16^int ^(arrow) cells are shown. In total, 16 healthy control (HC), 29 psoriasis (Ps), 29 psoriatic arthritis (PsA), and 8 rheumatoid arthritis (RA)patients were included in this analysis. See Table 1 for the summary of CD16^int ^percentage in HC, Ps, PsA and RA groups.

Based on the intermediate level of CD16 expression during osteoclastogenesis *in vitro *(Figure 2(b)-b), we assumed that a similar transition in CD16 expression might also occur *in vivo*. To explore this possibility, we examined CD16 expression on the same cohort of enrolled subjects referred to Figure 1(b) data (HC, n = 16; RA, n = 8; Ps, n = 29; PsA, n = 29). Intriguingly, we observed that PBMC of some subjects had a population with intermediate CD16 surface expression (Figures 3(a), b-d). Cells with intermediate expression of CD16 were designated as CD16^int^. Some subjects had only CD16+ and CD16- populations without CD16^int ^(Figure 3(a)-a). Although CD16^int ^cells were found in HC and patients, the frequency of individuals with CD16^int ^cells was significantly higher in patients than HC (86%, 67%, 61%, and 42% for Ps, PsA, RA, and HC, respectively; *P *= 0.01; columns b and c in Table 1). We further examined the expression of CD16^int ^cells in relation to CD14. Interestingly, CD16^int ^cells could be CD14- (arrow in Figure 3(b)-b) or CD14+ (arrow in Figure 3(b)-c). Collectively, our data provide direct evidence that up-regulation of CD16 does occur *in vivo *in human peripheral blood (Figures 3(a) and 3(b)), similar to the findings observed *in vitro *(Figure 2(b)). Therefore, it is conceivable that CD16^int ^cells represent a cell population undergoing a transitional state toward OC differentiation.

### CD16+ cells are the major reservoir of OCP in PsA patients

To determine if CD16+ cells are the reservoir for OCP, we sterile sorted CD16+ and CD16- cells and compared their osteoclastogenic potential. Due to a low absolute number of CD14+CD16+ cells in total PBMC (Figure 1(b) and column (a) in Table 1, range from 0.46 to 0.91%), we were unable to obtain enough sorted cells for OC culture *in vitro*. Because osteoclastogenesis is cell concentration dependent [23], cells cultured below a critical threshold density fail to encounter their fusion partners and thus cannot form multinucleated OC. To overcome the technical difficulty in obtaining enough cells for OC culture, we took two approaches. First, we obtained a large quantity of blood (about 400 ml) from hemochromatosis subjects undergoing therapeutic phlebotomy and sterile sorted CD14+CD16+ cells. Blood collected from hemochromatosis subjects was considered as controls in our study, because they were otherwise healthy without comorbid disorders. We were able to obtain enough CD14+CD16+ and CD14+CD16- cells for OC culture from these subjects after sterile sorting. The numbers of OC derived from 10^5 ^of the sorted CD14+CD16+ and CD14+CD16- cells were 40 ± 6 and 420 ± 38, respectively. This result is in accordance with the previous findings by Komano and colleagues [24] in which OC were derived from the CD16- subset in healthy individuals.

**Figure 4:**
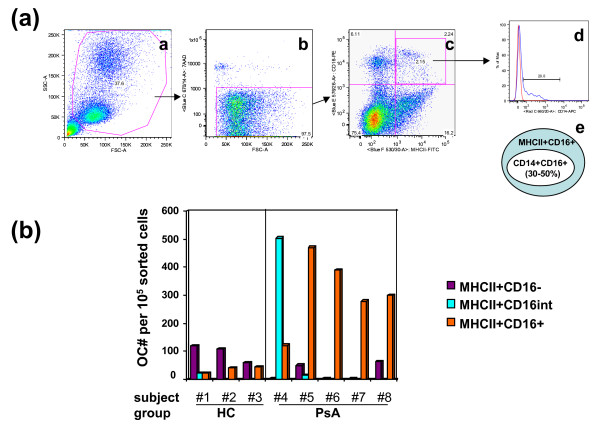
**CD16- and CD16+ cells are the major reservoirs of OC precursors in HC and PsA patients, respectively**. **(a) **30 to 50% of MHCII+CD16+ cells are CD14+. Human peripheral blood mononuclear cells (PBMC) were isolated from the peripheral blood and stained with an antibody cocktail composed of MHCII-FITC, CD14-APC, CD16-PE, 7AAD. The gating strategy is sequentially illustrated from a to d. [a] PBMC were gated by FSC/SSC; [b] live cells were gated by 7AAD; [c] dot plot analysis of MHCII and CD16 expression on live cells; [d] the expression of CD14 on MHCII+CD16+ cells gated from c. Red line is the isotype control and blue line is staining from CD14-APC. The number shown in the histogram represents the frequency of CD14+ cells per total MHCII+CD16+ cells. [e] The relative percentage of CD14+CD16+ to MHCII+CD16+ cells. Sample shown here is the representative of 3 healthy controls (HC). **(b) **Human PBMC from HC and psoriatic arthritis (PsA) patients were isolated, stained with antibodies, and sterile sorted into three populations, MHCII+CD16-, MHCII+CD16^int^, and MHCII+CD16+. Osteoclastogenesis was examined by osteoclast (OC) count after eight-day culture in the presence of receptor activator of nuclear factor kappa-B ligand (RANKL) and macrophage colony-stimulating factor (M-CSF) (MHCII+CD16-: purple, MHCII+CD16^int^: turquoise, MHCII+CD16+: orange). Subject numbers 1 to 3 are HC and numbers 4 to 8 are PsA patients.

Second, we chose to sort MHCII+CD16+ instead of CD14+CD16+ in order to obtain enough cells for OC culture. CD14+CD16+ cells are a subset (30 to 50%) of MHCII+CD16+ cells (Figure 4(a)-e). This conclusion was reached after examining the relation between MHCII+CD16+ and CD14+CD16+ using the following approach. Human PBMC were examined by an antibody cocktail composed of anti-CD16, anti-CD14, anti-MHCII antibodies and 7AAD (Figure 4(a)). Cells were first gated by FSC/SSC (Figure 4(a)-a), followed by dead cell exclusion with 7AAD (Figure 4(a)-b). MHCII+CD16+ double positive cells were gated (Figure 4(a)-c), and the percentage of CD14+ cells in total MHCII+CD16+ cells were analyzed (Figure 4(a)-d). Based on these data (Figure 4(a)-e), sorting of MHCII+CD16+ instead of CD14+CD16+ cells provided twice as many CD16+ cells forOC cultures.

PBMC from five PsA patients and three HC were sterile sorted following the same sorting parameters. Demographic data on these five patients is summarized in Table 2. The mean age of these five PsA subjects was 33 years, two had mild and three had severe PsA. They had six tender and swollen joints on average. Two of the subjects had dactylitis and one had axial disease. One subject had received an initial dose of infliximab four weeks prior to the blood draw but the other subjects were naïve to anti-TNF agents and DMARDs.

**Table 2:** Details of disease status for five PsA patients analyzed in Table 3 and Figure 4(b) at the time of sample collection

Patient number	Age	Gender	% BSA	Joint count
4	38	M	2%	9 T, S jts, 2 DD
5	40	F	4%	9 T, S jts, 2 DD
6	45	M	15%	4 T, S jts severe AD
7	27	M	80%	6 T, S jts
8	26	F	2%	8 T, S jts

AD = axial disease; BSA = body surface area; DD = dactylitic digits; F = female; jts = joints; M = male; PsA = psoriatic arthritis; S = swollen; T = tender.

Cells were sorted into three cell populations: MHCII+CD16-, MHCII+CD16^int^, and MHCII+CD16+. Sorted cells were cultured in the presence of RANKL and M-CSF for eight days, and TRAP-stained. Enumeration of OC from different sorted cell populations is summarized by Figure 4(b). Unexpectedly, OCP from HC and PsA patients arose from different cell subsets. In HC, OC were derived primarily from CD16- cells (Figure 4(b), subject numbers 1 to 3), whereas the majority of OC were derived from CD16+ or CD16^int ^cells in PsA patients (Figure 4(b), subject numbers 4 to 8). Although CD16+ cells were the major reservoir of OCP in most PsA patients (Figure 4(b), subject numbers 5 to 8, orange bars), it is noteworthy that CD16^int ^cells, instead of CD16+, were the source of OCP in one of the PsA subjects (Figure 4(b), subject number 4, turquoise bar). The results of cell sorting are shown in Table 3. Table 2 summarizes disease activity and patient information at the time of sample collection. Collectively, our results indicated that the major reservoirs of OCP in PsA patients and in HC are CD16+ cells and CD16- cells, respectively.

**Table 3:** Enumeration of OC derived from sorted MHCII+CD16- and MHCII+CD16+ cells in HC and PsA subjects

	MHCII+CD16-	MHCII+CD16+
	**OC# (per 10^5 ^cells)**	**OC# (per 10^5 ^cells)**

HC (n = 3)	97.6 ± 10.7	38.0 ± 4.1
PsA (n = 5)	25.8 ± 6.0	313.0 ± 26.1

HC = healthy control; OC = osteoclast; PsA = psoriatic arthritis.

**Figure 5:**
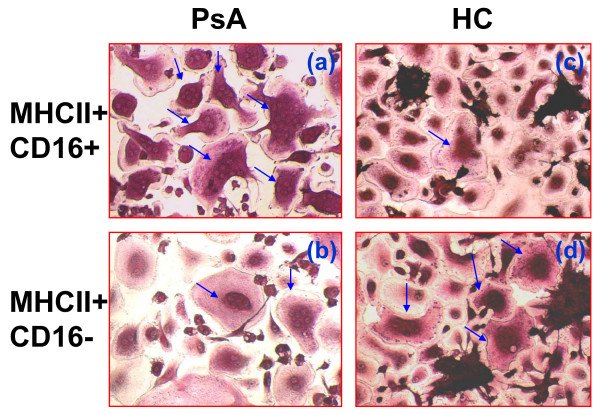
**OC derived from MHCII+CD16+ and MHCII+CD16- cells of HC and PsA patients have distinct phenotypes**. Peripheral blood mononuclear cells (PBMC) isolated from **(a and b) **psoriatic arthritis (PsA) and **(c and d) **healthy controls (HC) and were sterile sorted into (a and c) MHCII+CD16+ and (b and d) MHCII+CD16- populations. Sorted cells were cultured in the presence of receptor activator of nuclear factor kappa-B ligand (RANKL) and macrophage colony-stimulating factor (M-CSF) for eight days, and tartrate-resistant acid phosphatase stained for osteoclast (OC) quantification. Arrows indicate mature OC. The details of differences in OC generation potential and numbers of nuclei from these sorted subsets are summarized in Table 4.

Morphologic analysis provides additional support for the existence of divergent monocyte subsets with OC differentiation potential in PsA subjects compared with controls (Figure 5). OC derived from CD16+ monocytes in PsA subjects (Figure 5(a)) and CD16- cells cultured from HC (Figure 5(d)) were similar in regard to cell size and nuclear number. In contrast, OC derived from CD16- monocytes in PsA (Figure 5(b)) and CD16+ monocytes in HC (Figure 5(c)) displayed similar morphologic features. Furthermore, OCP from PsA subjects were larger and had more nuclei than those observed in controls. The relative cell diameter of OC derived from CD16+ cells in PsA was five times greater than the diameter observed in OC cultured from control CD16- cells (compare Figure 5(a) with 5(c)). The average number of nuclei per OC in PsA subjects was 12 ± 1.1 compared with 5 ± 0.2 in HC. The morphological differences in these cell subsets are summarized in Table 4.

**Table 4:** Summary of OC frequency and nuclear numbers in PBMC cultures derived from MHCII+CD16+ and MHCII+CD16- cell subsets in HC and PsA

	MHCII+CD16+	MHCII+CD16-
HC	30% of OC	70% of OC
	# of nuclei per OC = 3	# of nuclei per OC = 5 ± 0.2
PsA	90% of OC	10% of OC
	# of nuclei per OC = 12 ± 1.1	# of nuclei per OC = 3

Percentage of OC- and percentage of OC derived from a particular subject population (HC vs. PsA).

HC = healthy control; OC = osteoclast; PBMC = peripheral blood mononuclear cells; PsA = psoriatic arthritis.

### TNFα blocks CD16 expression on a subset of CD14+ monocytes

**Figure 6:**
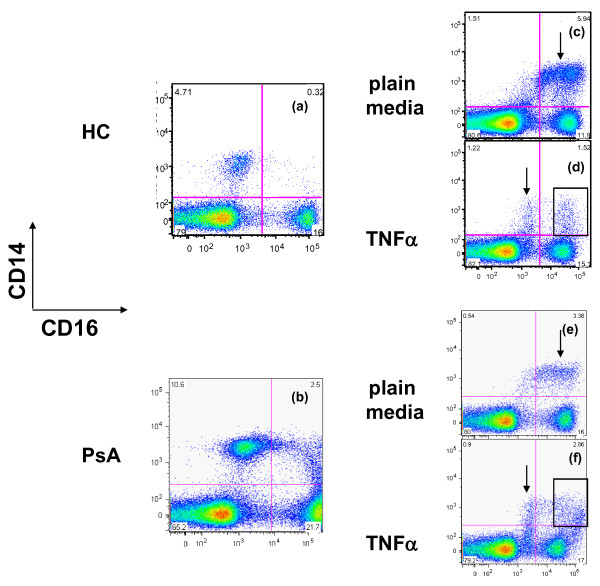
**CD14+cells from HC and PsA respond differently to TNFα in regards to CD16 cell surface expression**. The expression of CD16 on freshly isolated peripheral blood mononuclear cells (PBMC) from healthy control (HC) and psoriatic arthritis (PsA) was shown in **(a) **and **(b)**, respectively. After PBMC were cultured in the **(c **and **e) **absence or **(d **and **f) **presence of TNFα for three days, cell surface expression of CD16 was examined again using the same antibody cocktail composed of anti-CD16 (PE), anti-CD14 (APC), anti-CD3 (Pacific Blue), and anti-CD19 (APC-Cy7) antibodies with 7AAD. The anti-CD3, anti-CD19 antibodies and 7AAD were included to exclude T cells, B cells, and dead cells, respectively. Numbers shown in each quadrant (c to f) represent the percentage of cells per total gated live cells. Data shown here are the representative of two HC and three PsA samples.

The level of TNFα is elevated in psoriatic synovium and synovial fluid [3]. This finding coupled with the marked reduction of inflammatory signs and symptoms following anti-TNFα therapy suggest that TNFα plays a critical role in PsA pathology. Thus, it is plausible that elevated TNFα levels may account for the variations in OC ontogeny between HC and PsA shown in Figure 4(b). Of note, Skinner and colleagues reported that addition of TNFα to whole blood cultures of septic patients induced a marked expansion of CD14+CD16+ cells [25]. To examine this possibility, we analyzed the effect of TNFα on CD16 expression. Consistent to the data shown in Figure 1(b), fresh PBMC isolated from PsA subjects had a higher percentage of CD14+CD16+ cells than HC (Figure 6(b) vs. 6(a), upper right quadrants, 2.5% vs. 0.32%). In addition, the cell surface expression of CD16 in PsA was higher (compare the values of the x-axis on CD14+ cells, upper left and right quadrants, Figures 6(a) and 6(b)). After three-day culture in plain media, the percentage of CD14+CD16+ increased in both HC and PsA samples (0.32 to 5.94 for Figure 6(a) vs. 6(c), and 2.50 to 3.38 for Figure 6(b) vs. 6(e)), similar to the result after PBMC were cultured in OC-promoting conditions (Figure 2(b)).

Intriguingly, cells cultured in the presence of TNFα (Figures 6(d) and 6(f)) were more heterogeneous than cells cultured in the absence of TNFα (Figures 6(c) and 6(e)). It is likely that the presence of TNFα blocks CD16 upregulation on some CD14+ cells, resulting in two distinct CD14+ populations, CD14+CD16+ and CD14+CD16^int ^(indicated by vertical arrows in Figures 6(d) and 6(f)). In contrast, the majority of CD14+ cells upregulated CD16 expression and became CD16+ in the absence of TNFα (indicated by vertical arrows in Figures 6(c) and 6(e)). Interestingly, when exposed to TNFα, cells isolated from PsA patients had higher mean fluorescence intensities of CD16 expression than HC cells (compare CD16 expression on X-axis for CD14+CD16+ cells gated in rectangles, Figure 6(d) vs. 6(f)).

### Monocytes with a higher CD16 cell surface expression have increased bone resorption activity

The finding that the majority of OC in PsA patients were derived from CD16+ cells (Figure 4(b)); together with our previous result showing that elevated OCP correlate with joint erosions raised the possibility that the expression level of cell surface CD16 is associated with increased bone erosion activity. We examined this possibility with the bone wafer assay. MHCII+ cells isolated from three PsA patients were sterile sorted into CD16-, CD16^int^, and CD16+ populations and cultured together with bone wafers in the presence of RANKL and M-CSF. On day 14, bone wafers were TRAP-stained for OC quantification, followed by toluidine blue staining for visualization of erosion pits on the bone surface.

**Figure 7:**
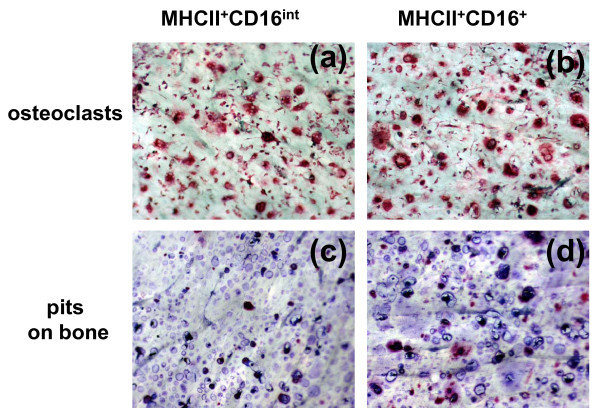
**Elevated CD16 surface expression was associated with increased bone erosion activity in PsA monocytes**. Sterile sorted MHCII+CD16+ and MHCII+CD16^int ^cells were co-cultured with bone slices in the presence of receptor activator of nuclear factor kappa-B ligand (RANKL) and macrophage colony-stimulating factor (M-CSF) for 14 days. **(a **and **b) **Osteoclast (OC) were identified by tartrate-resistant acid phosphatase (TRAP) staining, and **(c **and **d) **bone erosion pits were visualized after toluidine blue staining.

**Figure 8:**
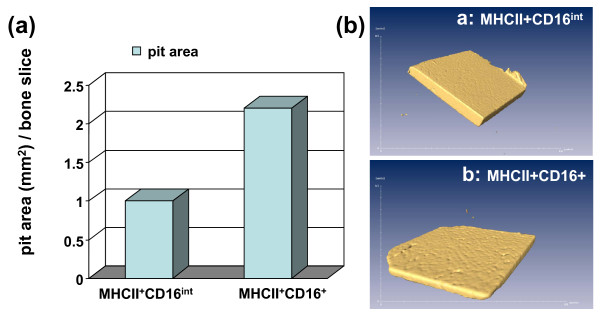
**CD16+ cells have a higher bone resorption activity than CD16^int ^cells**. Sterile sorted MHCII+CD16+ and MHCII+CD16^int ^cells were co-cultured with bone slices in the presence of receptor activator of nuclear factor kappa-B ligand (RANKL) and macrophage colony-stimulating factor (M-CSF) for 14 days. **(a) **Quantification of bone pits eroded by MHCII+CD16^int ^and MHCII+CD16+ cells. **(b) **Reconstructed three-dimensional micro-CT images of the bone wafer incubated with MHCII+CD16^int ^[a] and MHCII+CD16+ [b] sorted cells. The data are representative of three independent sorting experiments.

In contrast to MHCII+CD16- cells that generated very few TRAP+ cells on the bone wafer (data not shown), both MHCII+CD16^int ^and MHCII+CD16+ cells were able to develop into mature OC (Figure 7(a)-(b)). Figure 7(c)-(d) shows the corresponding bone erosions on the same bone wafers. OC derived from the CD16+ subset generated larger and more numerous pits than those from CD16^int ^cells (Figure 7(c) vs. 7(d)). Next, we used the method published by Zhang and colleagues [26] to quantify bone erosion area. The erosion area of the CD16+ cells was two-fold larger than that eroded by CD16^int ^cells (Figure 8(a)). We also noticed that the depth of erosion pits was also much deeper on the wafers incubated with CD16+ cells (Figure 7(c) vs. 7(d)). To confirm this observation, we used micro-CT technology to obtain three-dimensional images of these wafers (Figure 8(b)). In contrast to a moderate bone erosion observed in CD16^int ^sorted cells (Figure 8(b)-a), a higher number of pits associated with greater depth were found on the wafer incubated with CD16+ sorted cells (Figure 8(b)-b). In conclusion, our results showed that OC derived from CD16+ monocytes had a higher bone erosion activity than CD16^int ^and CD16- monocytes, which supports an association between CD16 surface expression and bone erosion activity.

## Discussion

Tissue macrophages are a diverse group of specialized cells that are pivotal in host defense, wound healing and immunoregulation [27]. They are thought to arise from circulating monocytes, which migrate to tissues and develop specific phenotypes such as Kupffer cells in the liver, microglial cells in the central nervous system and OC in bone [27,23]. Circulating monocytes are a heterogeneous population characterized by specific cell surface markers with unique proliferative and physiological properties that differentiate into specific effector cells in response to signals present in blood and tissues. Among these effector cells, OC are of particular interest due to the central importance of these cells in pathologic bone resorption in inflammatory arthritis. In previous studies, we demonstrated that the frequency of circulating OCP was greatly increased in a subset of PsA patients particularly those with joint destruction [3]. Herein, we show that PsA patients have an elevated percentage of CD14+CD16+ pro-inflammatory monocytes in the peripheral blood. Based on the observation of CD16 up-regulation in cells cultured in OC-promoting (M-CSF and RANKL) but not DC-promoting conditions (GM-CSF and IL-4), we examined whether CD16 up-regulation also occurs *in vivo *and identified cell subsets that expressed intermediate and high levels of CD16 in PsA. Intriguingly, we found OC arise from circulating CD16+ monocytes in PsA, whereas OC were generated from the CD16- subset in healthy controls. Finally, we showed a positive correlation between the level of CD16 cell surface expression and the extent of bone resorption. These studies indicate that the major reservoir of OCP in PsA is CD16+ cells, a finding that may catalyze the development of susceptibility biomarkers for arthritis in Ps patients and a treatment response marker in PsA patients with erosive arthritis.

In general, the CD14+CD16+ monocyte subset is thought to be more mature than the CD14+CD16- subset [6,7]. CD16^+ ^monocytes release high levels of pro-inflammatory cytokines and manifest phenotypic and functional characteristics of macrophages and DC [4,28]. Our results provide evidence that the CD16+ cells can also differentiate into OC. The existence of CD16^int ^cells with a lower level of bone resorption suggests that monocytes exhibit a transitional state in OC differentiation. Additional support for the plasticity of CD16 expression in these cells was the successful conversion of freshly sterile-sorted CD16- monocytes into CD16+ cells following overnight culture in media without exogenous cytokines (data not shown). Collectively, these data (Figure 2(b)) suggest that monocytes undergo a transition stage with intermediate expression of CD16 before differentiating into OC, presumably following exposure to RANKL and M-CSF in the bone marrow, circulation, and the joint. It is important to note that the clinical significance of CD14+CD16+ expansion may depend on the disease state. For example, although CD14+16+ cells are increased in psoriasis, inflammatory arthritis and sepsis, the differentiation fate of these cells is determined by cytokines and chemokines in the local microenvironment. It is likely that dendritic cells and inflammatory monocytes predominate in the case of sepsis, whereas osteoclastogenic cytokines foster OC differentiation in inflammatory arthritis.

Consistent with results of Komano and colleagues [24] and Lari and colleagues [29], our sorting results from HCs indicate that OC are derived from the CD16- monocyte subset. We were puzzled by our contradictory data in PsA subjects where the majority of OC were derived from CD16+ cells. One plausible explanation is that elevated levels of inflammatory cytokines and chemokines in PsA subjects promote the up-regulation of CD16 on monocytes. As a result, monocytes isolated from PsA subjects are more responsive to osteoclastogenic factors than those isolated from healthy individuals. Support for this concept was the finding that CD16 cell surface expression increased when cells were cultured with TNF or osteoclastogenic (RANKL + M-CSF) but not with DC-promoting cytokines (GM-CSF + IL-4) *in vitro*. Moreover, intermediate surface expression of CD16 noted in many Ps and PsA subjects may represent a transitional state in which monocytes are primed for osteoclastogenesis in response to environmental signals.

Although the importance of CD16 in immune regulation was emphasized by its critical role in uncontrolled systemic infection and sepsis [16,17], the function of CD16 in osteoclastogenesis remains largely unknown. Based on current available data, CD16 might be involved in the regulation of OC development through its ITAM. CD16 is considered an ITAM-bearing molecule due to its association with FcRγ [16,30]. The regulation of signaling through Fc receptors such as CD16 is extremely complex. With different affinities to ligand engagement, ITAM-containing Fc receptors generate either activating or inhibitory immune response signals [30]. Furthermore, cross-regulation and interaction between ITAM-associated receptors greatly magnifies the complexity of this regulation [31]. We propose a similar complex regulation of osteoclastogenesis by CD16 through its ITAM. To date, except for CD16, many ITAM-bearing surface receptors involved in OC differentiation have been well studied [32-34]. Humphrey and colleagues proposed a model whereby signals delivered by surface ITAM-bearing proteins regulate the expression of many genes involved in osteoclastogenesis [33]. This model provides a mechanism to explain the regulation of OC formation and provides an explanation for the positive correlation between the CD16 surface expression and bone erosion activity shown in this study. Current data suggest that ITAM-bearing proteins might act in concert to program cells into a fusion-competent state [35], regulate the multinucleation process [36], and recruit Syk kinase [33], similar to the model proposed by Humphrey and colleagues [33]. Understanding of the interactions between CD16 and other ITAM-bearing proteins will likely reveal the contribution of CD16 to osteoclastogenesis at the molecular level.

CD16 regulates the production of TNFα by both direct and indirect mechanisms. Binding of CD16 by *Escherichia coli *triggers TNFα secretion [16], and conversely, a dramatic decrease in TNFα production is observed in FcγIII-deficient mice [37]. Recently, Kramer and colleagues revealed how CD16 activation regulates TNFα production [38]. Activation of CD16 induces TNFα through the mitogen-activated protein kinase pathway but at the same time, CD16 activation can also limit TNFα production through phosphoinositide 3-kinase signaling. Of relevance to these findings is the fact that the proinflammatory CD14+CD16+ monocytes are major sources of TNFα, a cytokine that potentiates osteoclastogenesis [13], and IL-6, a cytokine that promotes OC maturation [39]. Therefore, it is likely that the elevated TNFα in PsA patients is partially responsible for the increased OCP in these subjects. Currently, we do not know if the expanded CD14+CD16+ cells are a major source of TNFα in PsA; however, it appears that TNFα does not induce CD14+CD16+ expansion through 'autocrine' regulation, because the frequency of CD14+CD16+ cells decreases in the presence of TNFα (Figures 6(c) and 6(e) vs. 6(d) and 6(f)). TNFα has a prominent inhibitory effect on CD16 cell surface expression for a subset of CD14+ monocytes (indicated by arrows in Figure 6(e) and 6(f)). This inhibitory effect of TNFα on CD16 expression was observed for all samples we processed including controls. Currently, it is unclear why TNFα blocks CD16 expression in a particular monocyte subset, resulting in two distinct CD14+ populations (Figures 6(d) and 6(f), upper left and right panels). It will be important to determine if the CD14+ cells with high CD16 expression are more likely to differentiate into OC compared with CD14+ cells with an intermediate level of CD16.

In conclusion, OCP derived from PsA patients display several unique properties compared with OCP that arise from HC, providing further support to our previous studies [3]. We showed that OCP arise from different monocyte populations in PsA subjects and healthy controls. In addition, TNFα upregulation of CD16 cell surface expression on CD14+CD16+ cells was significantly greater in PsA patients than in HC. We also demonstrated an expansion of circulating CD14+CD16+ monocytes in PsA subjects and identified cells that express intermediate levels of CD16. Moreover, surface expression of CD16 correlated with the extent of bone resorption *in vitro *for PsA monocytes. Collectively, our results suggest a model in which a subset of CD16- cells undergoes a transition to intermediate CD16 expression in response to inflammation, and subsequently differentiate into CD16+ cells prior to OC formation. Thus, CD16 may be a marker for OCP in PsA patients, although it is highly probable that additional molecules that specifically characterize this population will be identified. From a translational perspective, inhibition of the CD16- to CD16+ transition in circulating monocytes may have clinical applications for the treatment of metabolic and inflammatory bone disorders.

## Conclusions

In conclusion, we found an elevated frequency of circulating CD14+CD16+ cells in PsA subjects compared with HC. Moreover, the level of CD16 expression correlated with the bone resorption activity. We also demonstrated that many PsA PBMC cells expressed an intermediate level of CD16, and the expression of CD16 on fresh human monocytes was enhanced by osteoclastogenic cytokines. These data, together with the finding that OC were derived from the CD16+ population in PsA patients but not HC, suggest a model in which OCP undergo a transitional state characterized by a gradual increase in CD16 expression during osteoclastogenesis. Collectively, our data indicate that CD16 has the potential to serve as a marker of OCP in inflammatory arthritis.

## Abbreviations

7-AAD: 7-amino-actinomycin D; APC: allophycocyanin; DC: dendritic cells; DMARD: disease-modifying anti-rheumatic drug; FSC/SSC: forward scatter/side scatter; GM-CSF: granulocyte-macrophage colony-stimulating factor; HC: healthy control; Ig: immunoglobulin; IL: interleukin; ITAM: immunoreceptor tyrosine-based activation motif; M-CSF: macrophage colony-stimulating factor; OC: osteoclast; OCP: osteoclast precursor; PBMC: peripheral blood mononuclear cells; PBS: phosphate-buffered saline; PE: phycoerythrin; Ps: psoriasis; PsA: psoriatic arthritis; RA: rheumatoid arthritis; RANKL: receptor activator of nuclear factor kappa-B ligand; TNF: tumor necrosis factor; TRAP: tartrate-resistant acid phosphatase.

## Competing interests

The authors declare that they have no competing interests.

## Authors' contributions

YGC designed and performed most of the experiments, analyzed the data, drafted and finalized the manuscript. TS performed sterile cell sorting. CF performed statistical analysis. KAM and MT helped with micro-CT scanning on bone wafers. EMS provided scientific input and technical support. CTR designed and supervised the study, recruited patients, analyzed the data, edited and finalized the manuscript.
